# Utilization of antidepressants, anxiolytics, and hypnotics during the COVID-19 pandemic

**DOI:** 10.1038/s41398-024-02894-z

**Published:** 2024-04-04

**Authors:** Mikael Tiger, Giulio Castelpietra, Rikke Wesselhoeft, Johan Lundberg, Johan Reutfors

**Affiliations:** 1 Centre for Psychiatry Research, Department of Clinical Neuroscience, Karolinska Institutet & Stockholm Health Care Services, Region Stockholm, Sweden; 2 Outpatient and Inpatient Care Service, Central Health Directorate, Region Friuli Venezia Giulia, Trieste, Italy; 3 Department Adult 2, Centre Neuchâteloise de Psychiatrie, Marin-Epagnier, Préfargier, Switzerland; 4 Clinical Pharmacology, Pharmacy and Environmental Medicine, Department of Public Health, University of Southern Denmark, Odense, Denmark; 5 Research Unit of Child and Adolescent Psychiatry, Institute for Clinical Research, University of Southern Denmark, Odense, Denmark; 6 Centre for Pharmacoepidemiology, Department of Medicine Solna, Karolinska Institutet, Stockholm, Sweden

**Keywords:** Depression, Bipolar disorder

## Abstract

Since the onset of the COVID-19 pandemic, there have been concerns over the mental health impact of COVID-19. This is a review of the utilization of antidepressants, anxiolytics, and hypnotics since the COVID-19 pandemic was declared on March the 11th 2020. A number of reports so far have been based on large prescription databases for administrative use at the national or regional level, but mainly in high-income countries. We found studies reporting increased prescription rates of antidepressants, anxiolytics, and hypnotics during March 2020, which has been interpreted as hoarding of such medications. In the following months, most studies of antidepressant prescription rates did not display a clear pattern of change compared with prepandemic trends. In later phases of the pandemic small increases in utilization of antidepressants, with higher than predicted prescription rates, have been the most consistent finding, especially in youth. In most high-income countries, there were increasing trends in utilization of antidepressants also before 2020, which needs to be considered when estimating utilization during the pandemic, whereas for anxiolytics and hypnotics, the prepandemic patterns of prescriptions were more varying. Overall, after March 2020 we could not find any distinct changes in the utilization of anxiolytics and hypnotics during the COVID-19 pandemic. Most studies did not contain information about the prevalence of indicated psychiatric disorders in the studied populations. More studies are needed about the long-term effects of COVID-19, particularly regarding utilization of antidepressants. Research relating antidepressant utilization with the prevalence of major depression and anxiety disorders would promote a better understanding of how well antidepressant prescription rates reflect the needs of the population.

## Introduction

Since the onset of the global outbreak in early 2020, the coronavirus disease identified in 2019 (COVID-19) has profoundly impacted the world. On March 11, 2020, the World Health Organization (WHO) declared COVID-19 a pandemic. Three years into the pandemic, in May 2023, WHO stated that COVID-19 was no longer a public health emergency of international concern. China, the first country affected by the pandemic, implemented stringent lockdowns to control the spread of COVID-19, and many countries followed suit. Schools were closed, borders were, to varying degrees, closed, and various other strategies were employed to limit COVID-19 transmission [1]. Some countries, such as Sweden, relied on less restrictive measures and recommendations to the population [2]. With the disruptions to everyday life, coupled with the direct consequences of the disease itself, there have been concerns about the mental health impact of the pandemic. These concerns have led to calls for action to closely follow and mitigate the negative consequences of COVID-19 on mental health [3]. If the prevalence of depressive disorders, anxiety disorders, and sleep disorders would increase in the wake of the pandemic, we would anticipate increases in utilization of drugs to treat these conditions. The question of this review is: how has the COVID-19 pandemic affected the utilization of antidepressants, anxiolytics, and hypnotics?

### Antidepressants, anxiolytics, and hypnotics and their indications

Antidepressants are first-line medical treatments for anxiety disorders, unipolar and bipolar depression, the latter usually combined with mood stabilizers, such as lithium. Some antidepressants, such as mirtazapine, are also used as sedatives/hypnotics in clinical practice. Therefore, any correlation between changes in the prescription rates of antidepressants and the prevalence of specific disorders for which they are prescribed should be approached with caution. The term “anxiolytics” encompasses both benzodiazepines and “other anxiolytics”. Besides treating anxiety symptoms regardless of diagnosis, antihistamines, such as hydroxyzine, may be used for pruritus and urticaria. While benzodiazepines are mainly indicated for anxiety and sleep disorders, they are, to a lesser extent, prescribed for other conditions such as catatonia. Thus, when speculating about prevalent indications for anxiolytics and hypnotics based solely on prescription data, caution is warranted. Benzodiazepines are, in many countries, controlled substances and often require in-person visits, which could influence prescription rates during periods of social distancing.

### Incidence and prevalence of psychiatric disorders during the COVID-19 pandemic

Several studies have indicated an increase in mental health issues among children and adolescents throughout the COVID-19 pandemic [4–7], including sleep disturbances [7, 8]. Additionally, psychiatric disorders like depression [9] and anxiety disorders [9, 10] have presented a rise, particularly in studies spanning second lockdowns.

Similarly, adults have reported an increase in anxiety [11, 12] worldwide, and increased depressive symptoms have been reported in South Asia [12]. Prevalence rates of clinical depressive and anxiety disorders have also increased, particularly among females and younger age groups [13]. Subthreshold insomnia symptoms have increased globally, although moderate and severe self-reported insomnia symptoms remained relatively stable [14].

### Hoarding effects of medications during the COVID-19 pandemic

The COVID-19 pandemic has affected the overall utilization of drugs in various ways. One of the phenomena observed during the pandemic was medicine hoarding, which is the excessive accumulation of drugs for personal use or sometimes resale. Several studies have investigated drug hoarding during COVID-19, but they have not been able to disentangle this from stockpiling, which is the practice of acquiring and storing a supply of medications or drugs for legitimate reasons. In Sweden, there was an increase in the volume of dispensed drugs and over-the-counter drugs from mid-February 2020 to mid-March 2020, and the weekly rate of filled prescriptions for antivirals became historically high in this period [15, 16]. In a study of ten different drug groups in eight European countries/regions, there was an increase in the number of Defined Daily Doses (DDDs) for drugs for obstructive airway conditions during the COVID-19 period, and hoarding effects were seen across six countries/regions in March 2020 [17]. Comparing March 2020 to March 2019 in cross-sectional data of national drug purchases for 68 countries and jurisdictions, there was a 15% increase in the number of drug units purchased globally, reaching 5309.3 units per 100 population [18]. The increase was higher in high-income countries (18.5%) than in low-income countries (12.8%), however. The global purchase rate decreased after the March 2020 increase. Regarding psychotropic drug utilization during the COVID-19 pandemic, a study from Alberta in Canada [19] noted a surge in antidepressant dispensing from February 2020 to March 2021 compared to the preceding 13 months. Also, in five Canadian and US cities, 11.6% of people who used drugs reported drug hoarding in the prior month [20]. Significant increases in prescriptions for antidepressants, anxiolytics, and hypnotics were also observed in Northern Ireland during March 2020 compared to the previous years [21]. In Austria, there was a rise in prescribed DDDs for various psychiatric drugs before the first lockdown in March 2020, followed by a reduction in new patients initiating psychopharmacologic treatment during the first lockdown, though not during the second [22]. Since hoarding was widespread during March 2020, we will focus on utilization studies of antidepressants, anxiolytics, and hypnotics from April 2020 and onwards in the following sections.

### Utilization of antidepressants during the COVID-19 pandemic

The interplay between COVID-19 and antidepressant drug use is complex. On the one hand, infection with COVID-19 has been suggested to increase the risk of depressive and anxiety disorders [23], and mitigation measures such as school closures could affect mental health [24]. On the other hand, imposed social distancing could have reduced healthcare-seeking behavior and hence antidepressant drug utilization [25]. Moreover, in order to interpret changes in antidepressant drug utilization during COVID-19, prepandemic prescription rates and trends need to be considered. Countries with low antidepressant prescription rates may be more susceptible to changes related to major events such as the COVID-19 pandemic, while changes may be less evident in countries with higher prepandemic prescription rates. This could be illustrated by findings from late 2020 when antidepressant prescriptions increased in Denmark but not in Sweden, which had higher prescription rates than Denmark during 2015–2019 [26]. In most high-income countries, antidepressant drug usage has increased steadily during the past decade, with a few exceptions of relatively stable antidepressant drug prescription rates, such as in Denmark and Hungary [27]. Furthermore, selective serotonin reuptake inhibitors such as fluvoxamine have been tested to whether these drugs could reduce the risk of hospitalization for patients with COVID-19 [28]. Most of the studies on antidepressant drug utilization during the COVID-19 pandemic published so far were based on large prescription databases for administrative use at the national or regional level. The studies present results that roughly fall into one or two of the categories: increased, reduced, or unaltered utilization of antidepressants. Many reports on antidepressant use during the COVID-19 pandemic have demonstrated higher utilization than predicted from the prepandemic slope of antidepressant prescription rates. It seems that children and adolescents were more likely to have increased antidepressant utilization during the pandemic compared to adults [10, 26, 29, 30]. In a retrospective cohort study of a sample in Australia, the prevalent dispensing of antidepressants in children and adolescents was higher in 2021 than predicted, with the largest increase (22.2%) observed in girls [29]. In a study of national dispensing claims among citizens aged 10 years old and above in Australia during the first year of the pandemic, increased dispensing of antidepressants was limited to female youths, with increased prevalent and incident dispensing for girls aged 10–17 and increased prevalent dispensing in women who were 18–24 years old [30]. In all other age groups, as well as in males, antidepressants were dispensed as predicted in this interrupted time series study [30]. In a Danish register-based cohort study, increases in both incident and prevalent rates of filled antidepressant prescriptions among 5–24-year-olds were observed from March 2020 to June 2022 [10]. There was an 18% increase in incident users of psychotropic medication overall and a small (5%) increase in the incidence of psychiatric disorders during the pandemic [10]. An increase in antidepressant prescription fills in Danish and Norwegian youths (age 20 and younger) was observed in a register-based cross-country comparison in late 2020, while prescription fills remained within the predicted intervals in Swedish youths [26]. In Denmark, filled prescriptions were higher than predicted for all age groups at the end of 2020, while increased antidepressant utilization in Norway was limited to youth and unaffected by the pandemic in Sweden in December 2020, except for a small increase among female youth [26]. As in Denmark, utilization of antidepressants increased in France, with an increased slope of dispensed packages per population from March 2020 to September 2021, compared with that of the preceding five-year period [31]. In Israel, the pandemic pattern of antidepressant prescriptions was less clear-cut, as repeated lockdowns were associated with reduced utilization, although a trend of increased incident rates of antidepressant prescriptions was observed at the end of the study period, in January and February 2021 [32]. Likewise, in adolescents in Israel, higher than predicted incident antidepressant use was observed in 2021 based on data from the second-largest health maintenance organization in the country [9]. On a regional level, the Friuli Venezia Giulia Region in Italy had an increase in DDDs of antidepressants during the first year of the pandemic [33]. In addition to the utilization studies described above, a number of reports have not adjusted for the prepandemic trend of utilization. This potentially renders the findings inconclusive because an apparent increase may well be a simple extrapolation of the previous ascending pattern of prescription [34–40]. Some studies have reported reduced utilization of antidepressants during the first year of the COVID-19 pandemic. Before the availability of COVID-19 vaccines, the pandemic was mitigated by social distancing, ranging from recommendations to avoid crowding to strict lockdowns. At the same time, people were less inclined to seek health care for non-COVID-related complaints [41]. This is illustrated by a study of a large sample of Toronto-based medical records from primary care, where the visits for incident anxiety and/or depression and antidepressant prescription rates were transiently reduced in youth aged 10–18 years during April 2020 [42]. Similarly, Maguire et al. reported that fewer than predicted were prescribed an antidepressant in May 2020, ricocheting back to the predicted number of antidepressant users from June to October this year [21]. In this population-based study of registered prescriptions in Northern Ireland, a strong upward trend of psychotropic medication use was observed during the prepandemic period (January 2012 to February 2020) [21]. In Italy, the positive prepandemic slope of antidepressant utilization has not been so steep. Still, distinct reductions in incident antidepressant use were observed in relation to lockdowns in the Tuscany region in Italy [43]. In addition, there are a couple of studies showing reduced antidepressant utilization in 2020, which do not take the prepandemic patterns of prescription of antidepressants into account, thereby introducing a large degree of uncertainty regarding changes in utilization related to the pandemic [44, 45]. Unlike Denmark and Norway, Sweden did not have an overall increase in antidepressant prescription fills at the end of 2020 [26]. No lockdowns were imposed on the Swedish population [2], while the authorities in the neighboring Scandinavian countries implemented lockdowns, which was done twice in Denmark. However, the prepandemic antidepressant utilization was roughly twice as high in Sweden than in Denmark and Norway, which may have diminished any putative relative changes in antidepressant prescription fills in the Swedish population. However, antidepressant utilization remained within the predicted level during the pandemic period until October 2021, also in Poland [46], which has had an antidepressant utilization below the average of the European Union [47]. Thus, there are reports of changes in antidepressant utilization in all directions following the short period of medication stockpiling at the onset of the COVID-19 pandemic. The few studies observing reduced antidepressant use were all based on data from early into the pandemic and most likely reflect reduced mental health care utilization. Most studies report small increases in antidepressant utilization, which become more distinct throughout the pandemic period.

### Utilization of anxiolytics and hypnotics during the COVID-19 pandemic

Most of the aspects discussed for antidepressants can be applied to anxiolytics and hypnotics. It is further crucial to consider prepandemic prescription rates and trends for anxiolytics, where most reports are on the utilization of benzodiazepines (BZD) during the pandemic, and hypnotics to assess changes compared to predicted values. Similar to antidepressants, for instance, BZD and hypnotics prescriptions increased more in Denmark than in Sweden, which had higher prescription rates than Denmark during 2015-2019 [26].

Most of the studies on the use of BZD and hypnotics during COVID-19 were based on large national or regional prescription databases. Many of them overlapped with the studies on the use of antidepressants mentioned above since they were considering different psychotropics altogether. In line with studies on antidepressants, results fell into these rough categories: increased, reduced, or unaltered utilization.

Many studies reported a general increase in prescriptions of BZD and hypnotics after the start of the pandemic [19, 21, 26, 31, 34, 37, 40, 45, 48–51], and all took prepandemic prescription rates into account. Moreover, significant differences were observed between genders and age groups. A study from Northern Ireland showed a decreasing trend in prescriptions from 2012 until 2019 in hypnotics for all age groups and in BZD within the elderly only. In the first 8 months of the pandemic, however, hypnotic medication increased by 12% among young people, while utilization of anxiolytics was higher than expected in those aged 65 years and above [21]. A decreasing prepandemic trend in prescriptions of anxiolytics and hypnotics was also observed in France from 2015 to March 2020, followed by an increase in anxiolytic prescriptions 13 months after the COVID-19 outbreak [31]. Another French study observed that the extent of the increase of anxiolytics and hypnotics during the first 9 months of the pandemic was more pronounced in younger age groups (12-18 years and 19–25 years) when compared to the 5 previous years [51]. Similarly, there was an increase higher than predicted of hypnotics among young people in Nordic countries from March 2020, while this increase was less clear when considering BZDs [26]. Studies from different Spanish regions all reported an increase in BZD and hypnotics, albeit these studies used different methodologies and prepandemic periods for comparison [37, 45, 50, 52]. In Andalusia and Asturias, a more pronounced increase in utilization was found among the elderly and women [37, 52]. This was in line with a study from Portugal, where an increase in BZD was limited to adults aged 65 years and above [53]. In Italy, a moderate increase in anxiolytics was observed in the first months of the pandemic compared to 2019 [40]. Moreover, one study from Brazil and one from the US observed an increase only in hypnotics and not in anxiolytics [54, 55], more pronounced in women in the US [55]. Finally, two studies based only on young people reported an increase in anxiolytics and/or hypnotics during the pandemic. A Danish study based on young aged 14–22 years found an increase from March 2020 to June 2022 in the incident use of hypnotics, but not BZD, compared with the expected. This increase was paralleled by a rise in anxiety disorders demonstrated in the same study [10]. A study from the US found an increase in prescriptions of anxiolytics and hypnotics in children and adolescents receiving mental health services, but only at the onset of the pandemic, later returning to prepandemic levels [36]. There were also studies that found no changes in BZD and/or hypnotic prescriptions during the pandemic [46, 56–58]. The prepandemic period was analyzed in most of these studies with no changes in BZD and/or hypnotic prescriptions during the pandemic, showing different trends of prescriptions across areas: an increasing trend in Poland [46], a decreasing trend in Canada [57], and no change in the Scania region in Sweden during the time period before COVID-19 [58]. A single study considered different age groups and found no changes in BZD prescriptions during the pandemic in Canada. Interestingly, an Italian study [59] observed that BZD prescriptions varied across the pandemic periods when compared to the same prepandemic periods in 2019-2020. The study showed an initial decrease till July 2020, then an increase till September 2020 and finally a decrease from October 2020 to February 2021, which was paralleling the pandemic phases and related lockdowns. A Brazilian study [56] compared the use of BZD in the first quarter of 2021 with the first quarter of 2020, finding no significant increase during the pandemic. A study from Australia based only on young people with a maximum age of 18 years reported a decline in prescriptions of BZD and hypnotics from 2013 to 2021. No changes in anxiolytics and hypnotics prescribing during the pandemic were found when compared with the expected [29]. No changes were also found in the US in relation to the use of BZDs among children aged 0–19 years when comparing the last 9 months of 2020 with the same period of 2019 [60].

Four studies from North America found a decrease in benzodiazepines [55, 61–63], and three of them also observed a decrease in hypnotics. In all studies, prepandemic utilization trends were considered. A continuous decrease in BZD and hypnotics was found in California (US) from December 2017 to June 2020 [61], and only in BZD, considering all of the US [55]. A decrease in incident prescriptions of BZD, by 5.6% in 2020 compared with the expected, was also found in the US as a whole [63]. In Manitoba (Canada), there was a significant decline in incident prescriptions of anxiolytics and hypnotics compared to expected trends from March to December 2020 in most age groups and in both genders [62]. Finally, a study based only on young Finnish aged 14 to 22 years found a decrease in anxiolytics and hypnotics prescription rates compared to 2019. However, these patients showed a marked increase in seeking primary care for mental health problems in 2020 and 2021, particularly for sleeping disorders and anxiety [35].

To date, the literature does not show a clear increase in the utilization of anxiolytics or hypnotics during the pandemic. Nonetheless, it should be considered that prepandemic trends of these drugs mostly showed decreasing or unchanged prescriptions [21, 31, 55, 57, 58, 61, 62]. Further, the short extension of prepandemic periods has often hindered a broader picture of prescription trends over time, which might have led to a different interpretation of results. For instance, a French study found an increase in anxiolytics during 2020, which, however, did not reach the extent of prescriptions in 2015 [31].

## Conclusions and future directions

Despite the profound disruption caused by COVID-19, the influence of the pandemic on the utilization of antidepressants, anxiolytics, and hypnotics remains relatively modest, based on the available reports, which are limited to the first two years of the pandemic. Early in the pandemic, there was widespread hoarding of medications, including the stockpiling of antidepressants, anxiolytics, and hypnotics. However, after the first month, no consistent alterations in the utilization of these psychotropics were discerned, suggesting potential shifts between mental healthcare demand and reduced healthcare utilization due to COVID-19 mitigation measures. Notably, a trend towards increased utilization of antidepressants, particularly towards the latter stages of the pandemic, was observed. Nonetheless, it is important to contextualize these findings within the pre-pandemic prescription patterns, as many high-income countries had already seen substantial increases in antidepressant utilization before the pandemic struck. This was not sufficiently taken into account in some of the studies.

As yet, there is also a shortage of studies on the long-term effect of the pandemic on drug prescribing. The majority of published studies have been performed in high-income countries in Europe and North America, with only a few studies from Brazil and Australia. Additionally, most studies lack specific data on utilization by age, which is pertinent given the varied impact of the pandemic on different age groups. Furthermore, only a minority of utilization studies provide information on psychiatric disorders within the studied populations. Consequently, more comprehensive studies incorporating data on the incidence and prevalence rates of depressive, anxiety, and sleep disorders are needed to enhance our comprehension of the pandemic’s impact on mental health and to assess the extent to which prescription rates align with the needs of the population.
